# Are We Ready to Build Back “Healthier?” An Exploratory Analysis of U.S. State-Level Disaster Recovery Plans

**DOI:** 10.3390/ijerph18158003

**Published:** 2021-07-28

**Authors:** Mallory Kennedy, Shannon A. Gonick, Nicole A. Errett

**Affiliations:** Department of Environmental and Occupational Health Sciences, University of Washington, Seattle, WA 98195, USA; malloryjkennedy@gmail.com (M.K.); shannongonick@gmail.com (S.A.G.)

**Keywords:** disaster recovery, public health, plan content analysis

## Abstract

As communities recover from disasters, it is crucial to understand the extent to which states are prepared to support the recovery of health systems and services. This need has been emphasized by the United States’ experience with COVID-19. This study sought to assess public health activities in state disaster recovery implementation plans. In this exploratory, descriptive study, state-wide disaster recovery implementation plans were collected from emergency management agency websites and verified (*n* = 33). We reviewed and coded the recovery plans to identify health-related activities. While 70% and 64% of reviewed plans included activities to address short-term healthcare and behavioral health needs, respectively, one-third or less of the plans included activities to address long-term healthcare and behavioral health needs. Further, plans have limited descriptions of health-related data collection, analysis, or data-driven processes. Additional evidence-informed public health requirements and activities are needed in disaster recovery implementation plans. State disaster recovery plans would benefit from additional description of public health roles, responsibilities, and activities, as well as additional plans for collecting and analyzing public health data to drive recovery decision making and activities. Plans should include approaches for ongoing evaluation of recovery activities.

## 1. Introduction

The Federal Emergency Management Agency (FEMA) describes the National Preparedness Goal’s disaster recovery mission area as “capabilities necessary to assist communities affected by an incident to recover effectively,” and notes that recovery entails “timely restoration, strengthening and revitalization of infrastructure, housing and a sustainable economy, as well as the health, social, cultural, historic and environmental fabric of communities” [1]. FEMA’s National Disaster Recovery Framework (NDRF) highlights pre-disaster recovery plans as a means to provide a common platform for recovery decisions and actions [2]. In particular, recovery plans can help identify: roles, responsibilities, and opportunities for partnership; recovery priorities and policies; opportunities to incorporate hazard mitigation; and steps for post-disaster planning, processes, and coordination [2]. Therefore, disaster recovery plans may articulate and facilitate the execution of public health actions in recovery.

Recent work in the disaster recovery field has sought to further delineate health-related roles and activities in recovery. Key responsibilities of the health sector during disaster recovery include determining priorities for recovery; assessing recovery worker and community member health impacts and needs; supporting the restoration and provision of public health and healthcare services and systems; communicating with the public about available resources and services and health impacts and risks; collaborating with partners to support equitable recovery strategies; and conducting mitigation activities to improve resilience to future disasters and impacts of climate change [3,4,5,6,7]. Beyond advocating for recovery activities that are attentive to physical health, researchers have also emphasized a need to address mental and behavioral health during recovery, noting that supporting mental health is critical to the success of recovery strategies themselves [8].

Collaborative and dynamic planning processes and partnerships have also been identified as an essential aspect of public health preparedness [9] and community recovery [10]. The Public Health Emergency Preparedness and Response Capabilities developed by the United States Centers for Disease Control and Prevention (CDC) encourage public health agencies to partner with other organizations and community stakeholders during all stages of recovery [3]. Robust partnerships and a collaborative approach to disaster recovery can enhance and facilitate key recovery actions, including sharing resources and information, conducting ongoing needs assessments, and referring community members to services [11,12,13]. Studies based on real-world disaster recovery processes have also underscored the importance of partnerships and community engagement during disaster recovery; one case study found that undertaking a community-based participatory approach to providing public health services during disaster recovery facilitated the ability of the health sector to: identify, monitor, and address health needs and impacts in the community; connect community members to resources to support recovery; and contribute to the evidence base on health impacts of disasters [14]. Similarly, health sector leaders who participated in recovery from Hurricanes Irene and Sandy described including partners in recovery planning as a lesson learned based on their experiences [11]. In addition, a recent study based on Hurricane Sandy recovery found that partnerships between community-based organizations were associated with a greater perceived impact of recovery activities [13].

In addition to clarifying the role of the health sector during recovery, emphasis has been placed on the development of disaster recovery strategies that preemptively consider health impacts of strategies taken by other sectors (i.e., a health in all policies approach (HiAP)) [4]. The second edition of FEMA’s NDRF reflects these ideas and recommends including health considerations in recovery decision making, in addition to activities specific to responding to health needs and rebuilding health infrastructure [2]. Similarly, following the 2017 hurricane season, experts highlighted the opportunity to apply a HiAP approach to recovery [15,16]. A recently developed approach for measuring overall community recovery proposed a number of health-related indicators (i.e., indicators focused on restoring health services, providing mental health services, and performing environmental health functions), thus underscoring the importance of health activities in relation to overall community recovery [17]. However, a qualitative study to assess local health department engagement in Hurricane Harvey recovery identified impediments to applying a HiAP approach to recovery, including resource constraints and communication challenges [18].

Assessing states’ preparedness to incorporate health considerations in recovery is particularly timely and significant in light of the ongoing COVID-19 pandemic, due to its disproportionate effects on marginalized groups, widespread impacts to physical and mental health and the social determinants of health, and tremendous strain on health systems and workers [19,20,21,22,23,24,25]. As with recovery from other disasters, COVID-19 recovery has been identified as an opportunity to place health at the forefront by implementing recovery strategies that address the social determinants of health, build the resilience of health systems and communities, and promote health equity [25,26,27]. Proposed priorities for COVID-19 recovery include collecting and analyzing health data to identify ongoing impacts and needs, partnering with community- and faith-based organizations to deliver services, addressing mental health needs, focusing on the recovery of marginalized groups and the healthcare workforce, and identifying lessons learned and opportunities to improve the response to future events [28]. However, experts have highlighted potential barriers to recovery from infectious diseases and from COVID-19 specifically, including a relative lack of experience with large scale biological events, a tendency for recovery approaches to focus on restoring infrastructure rather than community health and well-being, longer-term health impacts and needs, and ongoing healthcare workforce and infrastructure issues [28,29].

Yet, it remains unclear whether and how guidance about integrating health considerations into recovery has been formalized in the context of state disaster recovery implementation plans. In order to assess the readiness of states to overcome the aforementioned barriers and integrate health considerations in disaster recovery, this exploratory, descriptive study sought to characterize health-related activities proposed in state disaster recovery plans and generate hypotheses that can be tested through future research.

## 2. Materials and Methods

State disaster recovery plans (developed or coordinated by state emergency management agencies, excluding agency-specific plans that are not part of an overall statewide recovery strategy) were collected from state emergency management websites in the summer of 2017. State emergency management agencies were contacted in the fall to confirm that the plan was the current and primary recovery plan, or to identify current plans if none were online. We attempted to collect or confirm plans through December 2017. A total of 34 plans were collected and confirmed. A total of 33 were coded and analyzed; one was excluded because it explicitly stated that recovery was outside of its focus. The remaining states failed to respond to our requests to confirm or obtain a copy of their plan, or did not have a recovery plan. No human subject data were obtained or analyzed for this study.

Two investigators (N.A.E. and S.A.G.) reviewed all 33 plans and developed a codebook and coding questions based on the recovery activity lists in the 2015 National Academy of Medicine (formerly the Institute of Medicine) Healthy, Resilient, and Sustainable Communities After Disasters report [4] and this familiarization process. See the Results section for an explanation of the categories from the codebook that guided our analysis.

Given the complexity of collecting health-related information or implementing health-related services and the associated need for specific planning, our analysis only captured explicit health-related activities/strategies and did not capture activities/strategies that may have an implied health component (e.g., assessments without mention of health agency/official involvement in design or implementation or health data collection). Given the lack of accepted recovery timelines, we deferred to the plans’ description of “short term” and “long term.” We considered activities short term unless the plan explicitly stated they were to be completed or continued in the long term.

Two investigators (N.A.E. and M.K.) reviewed and coded the following plan components for all 33 plans:

- Introduction, including mission/vision/purpose or equivalent;
- Plan organization (e.g., use of recovery support function (RSF) structure);
- Health-specific section of the plan (if applicable) or entire plan (if no health-specific section).

See Table 1 for a description of the specific sections reviewed for each plan.

We discussed and adjudicated all discrepancies in applied codes. NVivo qualitative analysis software (QSR International Pty Ltd., Victoria, Australia, Version 11, 2015 and Version 12, 2018) was used to code the plans and Microsoft Excel was used to capture responses to the coding questions and calculate descriptive statistics.

## 3. Results

State disaster recovery plans analyzed were formatted in a variety of ways, including as annexes to comprehensive emergency management plans, emergency support functions, or standalone emergency plans. A total of 39% (*n* = 13) of coded plans used an RSF format and 27% (*n* = 9) used the RSFs outlined in FEMA’s NDRF [2].

The majority of reviewed plans (70%) explicitly mention building back better in the mission/vision/purpose (or equivalent) of the overall plan, and approximately one-third (30%) explicitly mention health in the mission/vision/purpose (or equivalent) (Table 2). While 70% and 64% of plans include activities to address short-term behavioral health and healthcare needs, respectively, only one-third or less of the plans include activities to address long-term behavioral health and healthcare needs (Table 2).

Plans have limited descriptions of health-related data collection or analysis or data-driven processes. While most plans (67%) describe collecting or using health-related data in initial post-disaster recovery assessments, few plans reference utilizing pre-disaster health-specific assessments or data (15%), conducting ongoing health-specific assessments (27%), conducting surveillance activities (33%), or evaluating health-related recovery activities (9%) (Table 2).

## 4. Discussion

Our findings suggest that while some states have planned to implement a variety of health-related activities during recovery from disasters, including from COVID-19, there is a significant opportunity to improve the ability of recovery plans to facilitate health promotion in recovery. Templates, examples, and guidance may facilitate the development of recovery plans that more clearly articulate and facilitate the execution of public health roles in recovery. The National Association of County and City Health Officials’ (NACCHO) 2018 “Public Health Recovery Landscape Analysis”—a review of 21 local health department recovery plans/annexes and nine federal/national resources, and seven key informant interviews to better understand the state of local recovery planning—identified needs for publicly available plan examples, a unified command process for recovery, and clearer roles and responsibilities for different stakeholders in recovery [30], suggesting that such guidance and templates are also needed for local-level public health practitioners. NACCHO’s analysis also identified promising practices for local health department recovery planning, including language indicating transition from short- to long-term recovery, inclusion of checklists for health department recovery activities, and stakeholder engagement processes and advisory committee structures that align with the NDRF [30], that can be further refined through research and evaluation.

Characteristics of evidence-based public health include sound evaluation and systematic use of data and information systems [31]. As such, the lack of explicit intention to collect or analyze health-related data in state-wide recovery plans is particularly concerning, and may limit capacity to develop evidence-based practices for health promotion in recovery. It is possible that health activities may be informed by broader assessments (i.e., not specific to health) and may be evaluated in the context of overall recovery programs (i.e., in the context of broader after action reviews). As recovery may last for months or years, we suggest states plan for ongoing assessment, including identifying public health-specific assessment and evaluation questions, data streams, and approaches.

Similarly, recovery-related training may enhance public health workers’ understanding of and ability to plan for and implement health-related recovery functions. Free and publicly available online trainings, such as the National Center for Disaster Medicine’s Public Health System Training in Disaster Recovery, offer the potential for widespread access to recovery-related training [32]. Recovery-related programming at conferences (e.g., the Preparedness Summit) may help disseminate such trainings to public health preparedness practitioners.

The lack of attention to healthcare workforce issues and addressing long-term health needs in state recovery plans may also affect the success of COVID-19 recovery, given the pandemic’s widespread health effects and impacts to the healthcare workforce. Documentation of effective strategies and lessons learned related to recovering the healthcare workforce and addressing long-term healthcare and behavioral health needs during COVID-19 recovery could facilitate the inclusion of these areas in future disaster recovery planning efforts.

Our study has several limitations. First, this exploratory analysis is limited to state-wide disaster recovery plans, and further limited to specific sections of plans that were most likely to describe health-related recovery activities. It is possible that states or health departments have outlined implementation of health-related recovery activities in other plans; for example, in health department-specific plans or comprehensive emergency plans without specific recovery sections. In addition, we were only able to obtain and analyze plans from 33 states, meaning that plans from one-third of states were not included in our study. It is possible that the states that were able to confirm or provide plans had different characteristics than the states not represented in our analysis, so study results should be interpreted with this in mind. Additionally, as “community recovery” remains one of the core capabilities of the CDC’s Public Health Emergency Preparedness Cooperative Agreement [3], it is likely that states have endeavored to advance the public health role in recovery beyond what is captured herein. Finally, plans continue to evolve. We collected state recovery plans in 2017 and states may be in the process of updating or will update their plans, especially in light of extensive activities that will be required for COVID-19 pandemic recovery.

Future research should examine whether and how the COVID-19 pandemic motivated recovery planning and/or integration of health-related activities or requirements in recovery plans. Future studies could also assess the extent to which state approaches to COVID-19 recovery diverged from the actions prescribed by their pre-disaster recovery plans, evaluate the implementation and/or effectiveness of recovery strategies employed, and assess differences in planned recovery actions based on state characteristics, such as region, level of resources, or political leanings. Moreover, as the NDRF calls for local leadership in recovery [2], additional research is necessary to assess local-level plans for health promotion during recovery. Finally, future research should examine the quality of plans and appropriateness of strategies outlined, and assess other types of recovery plans that may contain additional health-specific information (e.g., recovery plans drafted by health departments).

## 5. Conclusions

Our findings suggest a need to improve the integration of health considerations in state disaster recovery planning. State disaster recovery plans could benefit from additional detail on health sector roles and responsibilities, data collection and analysis to inform recovery decision making and evaluate recovery strategies, strategies for addressing healthcare workforce issues, and approaches for providing long-term healthcare and behavioral health services. This study may also have implications for COVID-19 and other ongoing disaster recovery and the extent to which states are prepared to leverage recovery processes to promote population health, advance health equity, and strengthen resilience for future disasters.

## Figures and Tables

**Table 1:** State Disaster Recovery Plans Identified and Sections Analyzed.

State	Recovery Plan, Sections/Page Numbers (If Applicable), and Date (*n* = 33)
Alabama	-
Alaska	-
Arizona	Arizona Disaster Recovery Framework (updated 30 April 2014)-Introduction and Purpose Sections, pp. 2–7 and Recovery Support FunctionRSF Health and Social Services Appendix (updated 15 April 15 2014)
Arkansas	State of Arkansas Recovery Plan (updated 31 October 2014)
California	-
Colorado	Colorado Hazard and Incident Response and Recovery Plan, Base Plan pp. 1–27 and Support Function #8: Public Health, Medical Services and Behavioral Health pp. 1–12 (updated November 2016)
Connecticut	-
Delaware	Delaware Emergency Operations Plan, Recovery & Mitigation Branch, Long Term Recovery Group (updated February 2015)
Florida	State of Florida Recovery Plan, Recovery Annex to the State Comprehensive Emergency Management Plan (updated version approved 5 September 2013)
Georgia	Georgia Emergency Operations Plan: Emergency Support Function #14 Annex, Long-Term Recovery & Mitigation (updated 2015)
Hawaii	-
Idaho	Idaho Emergency Operations Plan: ID-ESF # 14 Long-Term Community Recovery and Mitigation (updated July 2015)
Illinois	State of Illinois Disaster Recovery Plan (updated June 2015)
Indiana	-
Iowa	Iowa Comprehensive Emergency Plan, Part C: State of Iowa Disaster Recovery Plan, Basic Plan 1.1–1.33 pp. 7–8 and Health and Social Services (RSF 3) pp. 65–76 (updated 1 February 2016)
Kansas	Kansas Response Plan ESF 14: Long Term Community Recovery (updated January 2017)
Kentucky	Kentucky Emergency Operations Plan ESF 14: Community Recovery (updated August 2014)
Louisiana	-
Maine	Emergency Operations Plan-Emergency Support Function 14: Transition to Recovery (updated 15 August 2016)
Maryland	State of Maryland Consequence Management Operations Plan, Base Plan pp. 10–46 and Recovery Chapter pp. 79–95 (updated September 2017)
Massachusetts	Massachusetts Comprehensive Emergency Management Plan, Massachusetts Emergency Support Function 14 (2013)
Michigan	Michigan Emergency Management Plan Recovery Support Plan (updated 11 June 2014)
Minnesota	Minnesota Disaster Recovery Assistance Framework (updated March 2010)
Mississippi	-
Missouri	-
Montana	Montana Emergency Response Framework, Base Plan pp. 2–23, Critical Infrastructure Restoration Considerations pp. 23–35, Individual and Family Services Considerations pp. 46–75, and Essential Government Services Considerations, pp 77–99 (updated January 2017)
Nebraska	-
Nevada	State Comprehensive Emergency Management Plan, Annex O—Emergency Support Function 14: Community Recovery (updated January 2014)
New Hampshire	New Hampshire Recovery Plan Chapter I Introduction pp. 5–11 & Recovery Support Function (RSF) 3 Health and Social Services Recovery pp. 45–49 (updated March 2015)
New Jersey	-
New Mexico	State of New Mexico All-Hazard Emergency Operations Plan, ESF #14: Long Term Recovery pp. 215–224 (Revised December 2016)
New York	New York State Comprehensive Emergency Management Plan, Volume 3: Long-Term Recovery Plan (updated March 2017)
North Carolina	North Carolina Disaster Recovery Framework, Introduction pp. 1–2 and Appendix 4 Health and Human Services A-4–1 (updated 18 October 2016)
North Dakota	Recovery Mission Area Operations Plan (updated September 25, 2017); Recovery Health and Social Services Branch Annex (updated 1 June 2016)
Ohio	Ohio Emergency Operations Plan, Emergency Support Function #14: Recovery and Mitigation (updated August 2017); State of Ohio Health and Social Services Recovery Strategy (updated July 2016)
Oklahoma	State of Oklahoma Long-Term Recovery Plan (updated 23 August 2017)
Oregon	-
Pennsylvania	ESF 14-Long-Term Community Recovery Annex (updated 2015)
Rhode Island	-
South Carolina	Appendix 6 (South Carolina Recovery Plan) to the South Carolina Emergency Operations Plan (updated October 2013)
South Dakota	-
Tennessee	Emergency Support Function 15: Recovery (updated 8 May 2015)
Texas	-
Utah	-
Vermont	State of Vermont Emergency Operations Plan, State Support Function (SSF) Annex 5: Emergency Management, Recovery & Mitigation (updated 12 May 2015)
Virginia	Commonwealth of Virginia Emergency Operations Plan: Emergency Support Function # 14: Recovery & Mitigation (updated August 2012); Commonwealth of Virginia Emergency Operations Plan: Support Annex #2 Recovery Programs (updated May 2015)
Washington	Emergency Support Function: ESF 14, Long-Term Recovery (updated May 2016)
West Virginia	State of West Virginia Emergency Operations Plan: Basic Plan (updated January 2016)
Wisconsin	State of Wisconsin Recovery Plan (updated May 2016)
Wyoming	-

**Table 2:** Health-Related Activities or Actions in State Disaster Recovery Plans (*n* = 33).

Plan Element	% (*n*)	Examples
Explicitly mentions building back better in the mission/vision/purpose (or equivalent) of the overall plan	70 (23)	“The overall focus of recovery is how best to restore, reconstruct, and redevelop the social, natural, and economic fabrics of the community, and encompasses more than the restoration of the community’s physical structures to their pre-disaster conditions.” “The priority for long-term recovery following major disasters in the state is to provide assistance to the affected local governments that will lead to restoring all essential services; repairing or replacing private and public property to pre-disaster condition; and, where possible, increase the community’s potential for a sustainable future.” “The Recovery Plan provides the functional framework for restoring impacted communities to their pre-disaster state with added resiliency.”
Addresses short-term behavioral health	70 (23)	Crisis counseling over the short term; spiritual or emotional care; Critical Incident Stress Debriefing
References conducting/collecting or utilizing post-disaster health-specific assessments or data	67 (22)	Community Assessment for Public Health Response (CASPER) or other community health/community health needs assessment; behavioral crisis call center data; impact analysis of health system
Addresses short-term healthcare	64 (21)	Patient evacuation and emergency or temporary medical assistance, including medications; Other Needs Assistance (ONA) reimbursement for medical and dental costs; reconnecting affected individuals with health services; procuring medical supplies (e.g., blood, medications, medical devices)
Addresses health system restoration	58 (19)	Temporary restoration of critical infrastructure (inclusive of hospitals and other healthcare facilities); rebuilding health systems to pre-disaster levels or better; collaboration with other entities around health system restoration; Certificate of Need application process for healthcare facilities
Addresses long-term behavioral health	33 (11)	Crisis counseling over the long term; ongoing psychological/emotional support
Describes conducting human health-related surveillance activities	33 (11)	Deployment of epidemiological teams to monitor health needs; ongoing epidemiological surveillance of affected or displaced communities; shelter surveillance
Explicitly mentions health in the mission/vision/purpose (or equivalent) of the overall plan	30 (10)	“The long-term recovery of key health, social, cultural, historic, and environmental components of our state.” “Recovery includes those activities that enable people and organizations from impacted jurisdictions to restore, redevelop, and revitalize the health, social, economic, natural, and environmental fabric of the community, and to plan long-term actions to build resilience and mitigate the effects of future disasters.” “The Framework is based on the premise that the top priorities during disaster recovery are: public health and safety, protection of property, and the restoration of the economic vitality of the disaster area.”
Addresses healthcare workforce issues	30 (10)	Medical volunteer licensing, credentialing, and management; healthcare recovery worker training; Disaster Medical Assistance Teams; Medical Reserve Corps
References conducting/collecting or utilizing ongoing health-specific assessments	27 (9)	Ongoing assessments of health needs; ongoing assessments of health-related resources (e.g., medication)
Addresses long-term healthcare	24 (8)	Continued delivery of short-term healthcare services in the long term; technical assistance/expertise on long-term healthcare needs and plans for service delivery
References conducting/collecting or utilizing pre-disaster health-specificassessments or data	15 (5)	Extant health services support needs data; data on the health services system’s pre-disaster condition; health and behavioral health service enrollment data; immunization data
References evaluating health-related recovery activities	9 (3)	“Evaluation of the effectiveness of public health activities (i.e., are needs being met, is the community receiving appropriate messaging, are implemented programs successful?)”

## Data Availability

Data are publicly available and were obtained from state emergency management agencies or their websites, and are available through these agencies, their websites, or the authors.
